# Comparative transcriptome analysis of different chemotypes elucidates withanolide biosynthesis pathway from medicinal plant *Withania somnifera*

**DOI:** 10.1038/srep18611

**Published:** 2015-12-21

**Authors:** Parul Gupta, Ridhi Goel, Aditya Vikram Agarwal, Mehar Hasan Asif, Neelam Singh Sangwan, Rajender Singh Sangwan, Prabodh Kumar Trivedi

**Affiliations:** 1 CSIR-National Botanical Research Institute (CSIR-NBRI), Rana Pratap Marg, Lucknow-226 001, INDIA; 2 CSIR-Central Institute of Medicinal and Aromatic Plants (CSIR-CIMAP), Lucknow-226015, INDIA; 3 Academy of Scientific and Innovative Research (AcSIR), Anusandhan Bhawan, 2 Rafi Marg, NewDelhi-110001, INDIA

## Abstract

*Withania somnifera* is one of the most valuable medicinal plants synthesizing secondary metabolites known as withanolides. Despite pharmaceutical importance, limited information is available about the biosynthesis of withanolides. Chemo-profiling of leaf and root tissues of *Withania* suggest differences in the content and/or nature of withanolides in different chemotypes. To identify genes involved in chemotype and/or tissue-specific withanolide biosynthesis, we established transcriptomes of leaf and root tissues of distinct chemotypes. Genes encoding enzymes for intermediate steps of terpenoid backbone biosynthesis with their alternatively spliced forms and paralogous have been identified. Analysis suggests differential expression of large number genes among leaf and root tissues of different chemotypes. Study also identified differentially expressing transcripts encoding cytochrome P450s, glycosyltransferases, methyltransferases and transcription factors which might be involved in chemodiversity in *Withania*. Virus induced gene silencing of the sterol ∆7-reductase (*WsDWF5*) involved in the synthesis of 24-methylene cholesterol, withanolide backbone, suggests role of this enzyme in biosynthesis of withanolides. Information generated, in this study, provides a rich resource for functional analysis of withanolide-specific genes to elucidate chemotype- as well as tissue-specific withanolide biosynthesis. This genomic resource will also help in development of new tools for functional genomics and breeding in *Withania*.

*Withania somnifera* is a rich repository of secondary metabolites, which include several bioactive alkaloids and steroidal lactone based phytochemicals. The pharmacological activities of *W. somnifera* include anti-osteoporotic, anti-arthritic, anti-aging, anti-cancer, physiological and metabolic restorative properties as well as recovery from neurodegenerative disorders^1^,^2^,^3^,^4^,^5^,^6^. These medicinal properties of *W. somnifera* are attributed to the active key constituents named withanolides and their glyco-conjugates (glycowithanolides) present in different plant parts^7^,^3^,^8^,^9^.

Withanolides, pharmacologically active constituents from *Withania*, are a group of molecules having C-28 steroidal backbone in which C-26 and C-22 or C-26 and C-23 are oxidized to form δ- or γ-lactone ring respectively^10^. Chemical structures of different withanolides suggest that diversity is generated due to hydroxylation, glycosylation, formation of additional rings and addition of the side chains in this steroid backbone. Studies suggest that precursor molecules for the withanolide biosynthesis are isoprenoids, synthesized via classical cytosolic mevalonate (MVA) and plastid localized 2-C-methyl-D-erythritol-4-phosphate (MEP) pathways leading to biosynthesis of 24-methylene cholesterol (C_30_ terpenoid)^11^,^12^,^13^,^14^. Metabolic divergence from the sterol pathway, at the level of 24-methylene cholesterol, leads to formation of different withanolide moieties. Major withanolides with pharmaceutical activities include withaferin A, withanolide A, withanone and withanolide D in chemotype- and/or tissue-specific manner^15^.

Due to immense importance of *W. somnifera* in ancient and modern medicinal systems, this plant has been selected under sequencing of 100 genomes of *Solanaceae* plants in Sol Genomics Network (http://solgenomics.net/organism/sol100/view). Studies suggest that different chemotypes and tissues of *Withania* synthesize few specific molecules^16^. Therefore, generation of EST datasets of the different tissues/ chemotypes may provide information about gene expression profiles in specific tissues/ chemotypes as well as help in establishing biosynthesis of specific withanolides. In past, using Next-Generation Sequencing (NGS) a number of transcriptome datasets have been developed and used for discovery and prediction of genes involved in the secondary metabolite biosynthesis from various medicinally important plants^17^,^18^,^19^,^20^,^21^,^22^. Recently, transcriptome sequence of leaf and root tissue of one of *Withania* chemotype (NMITLI-101) was established^23^. However, this limited information (only one chemotype) restricts the understanding of the chemotype-specific withanolide biosynthesis as well as its correlation with chemo-diversity in *Withania*.

In this study, to unravel the biosynthesis of specific withanolides transcriptome datasets of leaf and root tissues of distinct chemotypes have been established. Datasets developed, in this study, from different chemotypes as well as of NMITLI-101 chemotype^23^ have been used for comparative gene expression analysis. Analysis established sequences of genes encoding enzymes catalyzing various intermediate steps of withanolide biosynthetic pathway as well as contribution of the 24-methylene cholesterol in the biosynthesis of withanolides using Virus Induced Gene Silencing (VIGS). In addition, a number of genes involved in the secondary conversion for the biosynthesis of specific withanolides have been identified. The genomic resource, genes linked to chemodiversity and tools for functional genomics, developed in this study, will help in establishing withanolides biosynthetic pathway and pave a way towards enhanced biosynthesis of the specific withanolides through synthetic biology approach.

## Results

### Analysis of major metabolite content in different chemotypes

**Figure 1:**
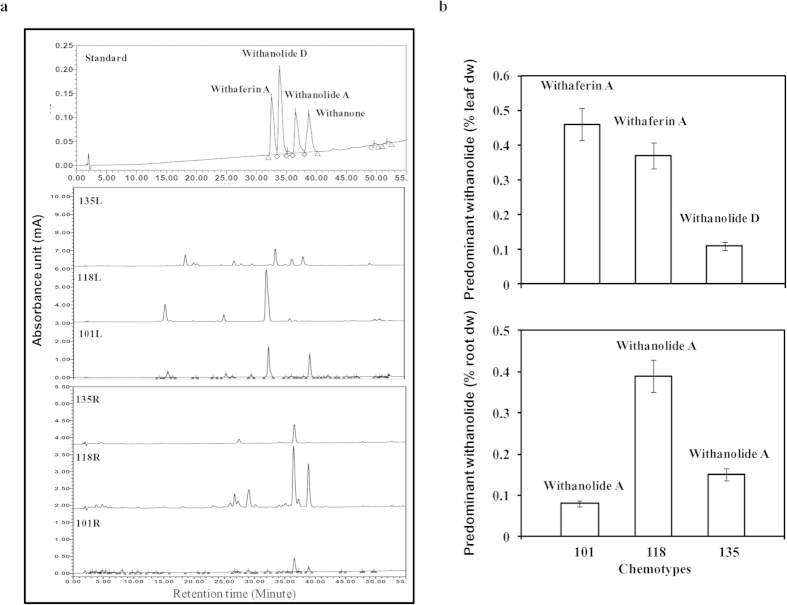
Differential withanolide biosynthesis in leaf and root tissues of three different chemotypes. (**a**) Representative HPLC profiles of NMITLI-101 (leaf: 101L; root: 101R), NMITLI-118 (leaf: 118L; root: 118R) and NMITLI-135 (leaf: 135L; root: 135R) with specific withanolides. (**b**) Predominant withanoldes in leaf and root tissues of three different chemotypes.

Phytochemical analyses of leaf and root tissues of different chemotypes (Fig. 1a) suggested that withaferin A as well as withanone are the main withanolides in leaf tissue of NMITLI-101. In NMITLI-118 and NMITLI-135, withaferin A and withanolide D are the main withanolides, respectively (Fig. 1b). Maximum withaferin A accumulated in NMITLI-101 (4.6 μg/mg leaf dry weight). In case of root tissues, withanolide A was predominantly found as main withanolide in all three chemotypes (Fig. 1b). Withanolide D accumulated in leaf (1.1 μg/mg leaf dry weight) tissues of NMITLI-135 and was not detected in NMITLI-101 and NMITLI-118.

### Establishment of *Withania* transcriptomes

In previous study, we reported leaf and root transcriptomes of NMITLI-101 chemotype^23^. To study chemotype-specific as well as tissue-specific withanolide biosynthesis, long-read transcriptome sequencing from leaf and root tissues of two other chemotypes (NMITLI-118 and NMITLI-135) was carried out. Sequencing run of leaf and root tissues of NMITLI-118 yielded 6,75,691 (210 Mb) and 7,31,352 (226 Mb) of HQ ESTs respectively. Similarly, 7,08,367 (207 Mb) and 5,98,182 (172 Mb) high quality ESTs were generated from leaf and root tissues of NMITLI-135 respectively. The sequences from all the libraries were deposited at NCBIs Short Read Archive under the accession number SRA 101323 (leaf run no. SRR1019197 and root run no. SRR1019198) for NMITLI-118 and SRA 106117 (leaf run no. SRR1012863 and root run no. SRR1012864) for NMITLI-135. Details of the sequencing data generated, in this study, and used for analysis is provided in the Supplementary Table S1.

### *De novo* assembly and annotation

Assembly of reads obtained from NMITLI-118 library resulted into generation of 20,621 and 22,438 contigs as well as 63,910 and 78,645 reads remained as singletons from leaf and root tissue, respectively. In case of NMITLI-135 leaf tissues, 70,8367 reads assembled into 20,135 contigs and 66,176 reads remained as singletons. Similarly, 5,98,182 reads were assembled in 18,413 contigs and unassembled reads remained as 79,737 singletons from NMITLI-135 root library. Average length of contigs in all the libraries was in the range of 599 bases to 627 bases. The size distribution of the raw reads and assembled contigs from different libraries are represented in Supplementary Fig. 1. Among all the contigs, 45% to 52% contigs were considered as the large contigs with average length ranges between 889–925 bases in leaf and root libraries of both the chemotypes. GC content of the assembled contigs and singletons in leaf and root tissues of both chemotypes ranged between 41 to 42% and 39 to 40.36%, respectively (Supplementary Table S1).

Annotation of contigs and singletons generated from assembly of reads from NMITLI-118 and NMITLI-135 leaf and root libraries was carried out using BLASTx against TAIR10, NCBI (NR) and tomato genome databases. BLAST annotation of large number of contigs and singletons from NMITLI-118 and 135 as well as previously reported NMITLI-101 indicated extensive coverage of *W. somnifera* transcriptomes. In 118L and 135L, a total of 35,510 and 35,493 unigenes and from 118R and 135R, a total of 38,383 and 38,047 unigenes were annotated against TAIR10 database. BLASTx results against NCBI (NR) database annotated 38,666 and 38,557 unigenes from 118L and 135L, respectively. In case of root tissues of 118 and 135 chemotypes, 43,116 and 42,844 unigenes were annotated against NCBI (NR) database. BLASTx results against the tomato genome database provided 45,088 and 45,818 annotated unigenes from 118L and 135L respectively. Similarly, from 118R and 135R, a total of 31,376 and 49,068 unigenes were annotated against the tomato genome database respectively. Out of three different databases, maximum annotation for each library was observed using tomato genome database. It was observed that more than 48% unigenes from different datasets, including NMITLI-101, were annotated against the tomato genome database. Among three different databases, 34,400 and 37,116 unigenes were annotated commonly in leaf and root tissues of the NMITLI-118 chemotype (Supplementary Fig. 1c, d). Similarly, in leaf and root libraries of the NMITLI-135, total 34,269 and 36,596 unigenes were annotated commonly from three different databases (Supplementary Fig. 1g, h). In all three chemotypes, maximum number of uniquely annotated unigenes was observed using tomato genome database, which indicated that being a Solanaceae plant, transcripts from *Withania* have higher homology with the tomato genome.

### Features of the combined assembly

**Figure 2:**
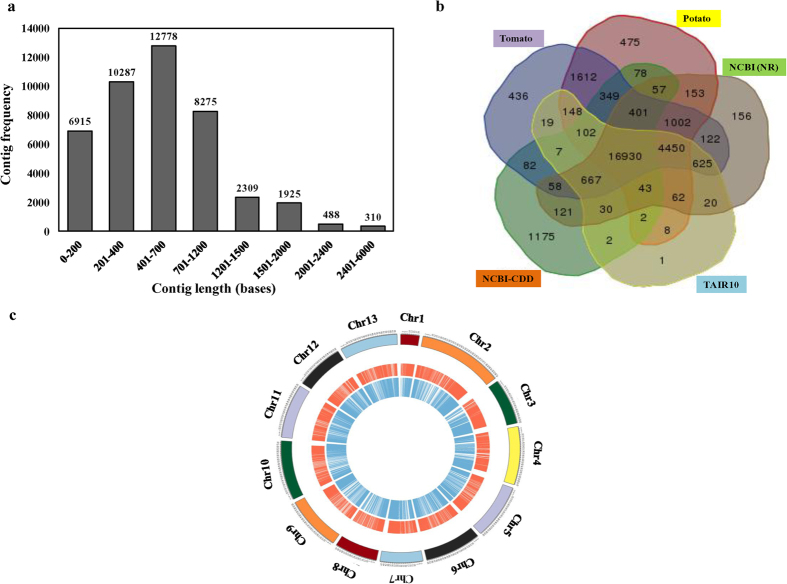
General features of *Withania* transcriptome. (**a**) Size distribution of assembled contigs from the combined assembly. (**b**) Common and unique annotation of contigs from combined assembly against different databases. (**c**) Synteny plot representing tomato chromosomes (1–13), tomato transcriptome (red circle) and unigenes (contigs+singletons) generated from *Withania* transcriptome (blue circle).

To identify gene families leading to chemodiversity in all three chemotypes, contigs and singletons obtained from assembly of each transcriptome including 101L, 101R^23^, 118L, 118R, 135L and 135R were tagged, pooled and assembled again using 454 GS Assembler. It has been considered that the super assembly of all primary assemblies generated using different programs gives better results in terms of consistency and size of contigs as well as alignment to the reference sequences^24^. This analysis was carried out to get larger contigs and to study digital differential expression of different unigens in different tissues of the chemotypes. Combined assembly using six transcriptomes resulted into 43,287 contigs however 1,78,956 reads\contigs remained as singletons (Supplementary Table S2). Length of contigs ranged between 100–5278 bases with an average length of 617.05 bases. There were 36,372 contigs which were of more than 200 bases length. The size distribution of assembled contigs is represented as Fig. 2a. Among all the contigs, 48% contigs were considered as the large contigs with average length ranges of 973 bases. GC content of the assembled contigs and singletons was 40.38% and 38.00%, respectively. Length of the singletons ranged in between 50–1967 bases with average length of 258.28 bases.

Annotation of the contigs generated from the combined assembly of three different chemotypes of *Withania* was carried out using BLASTx against TAIR10, NCBI (NR), potato genome database, tomato genome databases and NCBI-CDD (Supplementary Table S3). A total of 23,116 contigs were annotated using TAIR10 where as 24,897, 25,872, 27,010 and 20,104 contigs were annotated using NCBI-NR, potato genome database, tomato genome database and NCBI-CDD, respectively (Fig. 2b). Out of these different databases, maximum annotation was observed using tomato genome database followed by potato genome database. Of the total contigs assembled, 16,930 contigs were annotated commonly from different databases. A synteny was plotted using 13 chromosomes of tomato (including one pseudochromosome), tomato CDS and contigs as well as singletons generated from the combined assembly of all the six *Withania* transcriptomes to understand similarity between *Withania* transcriptome and tomato genome (Fig. 2c), as both the plants belong to same taxonomic family. This analysis shows that *Withania* transcriptome is similar to tomato transcriptome/genome and its gene homologs are spread over to the different chromosomes of tomato.

### Gene Ontology classification

**Figure 3:**
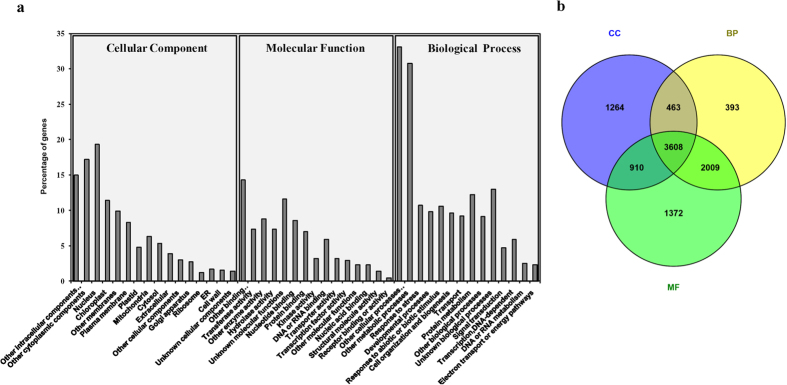
Histogram of gene ontology classification. (**a**) Classification of the unigenes in three main categories: cellular component, molecular function and biological process. Bars represent the percent number of assignments of contigs generated from the combined assembly with BLAST matches in the TAIR10 database to each GO term. (**b**) Common and unique contigs assigned in one or more than one GO categories.

GO assignments were used to classify the functions of unigenes generated from the combined assembly of all the six transcriptomes. Based on sequence homology, all unigenes were categorized into 45 functional groups (Fig. 3a). In each of the three main categories (cellular component, molecular function and biological process) of the GO classification, ‘other cellular process’ and ‘other metabolic process’ terms were dominant (≥30%), respectively. High percentage of unigenes from categories of ‘nucleus’ ‘other cytoplasmic components’, ‘other binding’, ‘other intracellular components’, ‘unknown biological process’ and ‘protein metabolism’ were classified. Extremely low percentage of the genes were classified in terms of ‘receptor binding or activity’ category. A large number of the unigenes were designated to ‘other metabolic processes’, which suggests that study may allow for the identification of novel genes involved in the secondary metabolite biosynthesis pathways from *W. somnifera*. Out of total unigenes categorized in three main categories, 3608 unigenes were commonly assigned in the cellular component, molecular function and biological process categories (Fig. 3b). Maximum annotation (1372 unigenes) was observed in the molecular function category (Fig. 3b).

### Functional characterization using KEGG

To identify the biological pathways which are functional in *W. somnifera*, 2,22,240 annotated sequences (contigs and singletons) from the combined assembly of all six transcriptomes were mapped to the reference canonical pathways in KEGG. In total, all contigs and singletons were assigned to 124 KEGG pathways. A total of 7,830 unigenes were found to be involved in biosynthesis of various secondary metabolites (Table 1). Out of all the secondary metabolic pathways, the cluster for ‘Phenylpropanoid biosynthesis [PATH: ko00940]’ represents the largest group (225 members) followed by ‘Stibenoid diarylhepatanoid and gingerol biosynthesis [PATH: 00945] (111 members) and ‘Limonene and pinene degradation [PATH: ko00903]’ (103 members). ‘Terpenoid back bone biosynthesis [PATH: ko00900]’ included 102 members.

### Identification of differentially expressed genes

**Figure 4:**
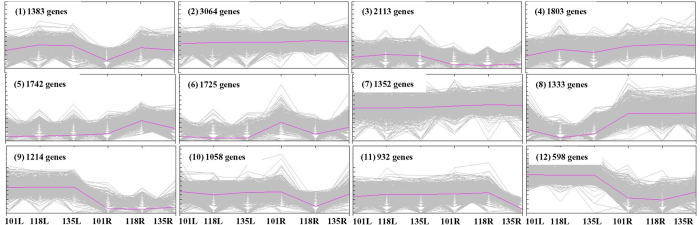
Clustering of genes on the basis of expression pattern. Differentially expressed genes in leaf (L) and root (R) tissues of NMITLI-101, NMITLI-118 and NMITLI-135 were used for the analysis. Each cluster represents a set of differentially expressed genes. Bold lines in different clusters represent collective expression pattern of all the genes present in each cluster.

Identification of differentially expressed genes was carried out on the basis of R value ≥12. Out of 43,287 contigs generated from the combined assembly, a total of 18,317 contigs were identified as differentially expressed in leaf and root tissues of three different chemotypes. Unigenes, involved in generation of larger contigs in combined assembly, were only considered for digital differential expression analysis and hence singletons were excluded. Out of 18,317 contigs, 13,176 were annotated using different databases. Number of commonly annotated contigs was 11,272 whereas 142, 6 and 943 differentially expressed contigs were annotated uniquely against NR, TAIR10 and tomato genome database, respectively (Supplementary Fig. 2). Expression pattern of all differentially expressed genes in leaf and root tissues of three different chemotypes were represented in 12 clusters. Each cluster shows variable number of differentially expressed genes (Fig. 4). Among all 12 clusters, genes which expressed highly in leaf tissues as compared to roots of all three chemotypes were clustered in cluster 9 and 12. Similarly, genes with higher expression in roots as compared to leaves were gathered in cluster 8. Genes present in cluster 2 and 7 showed almost equal expression in leaf and root tissues of three different chemotypes.

### Genes involved in terpenoid backbone biosynthesis

**Figure 5:**
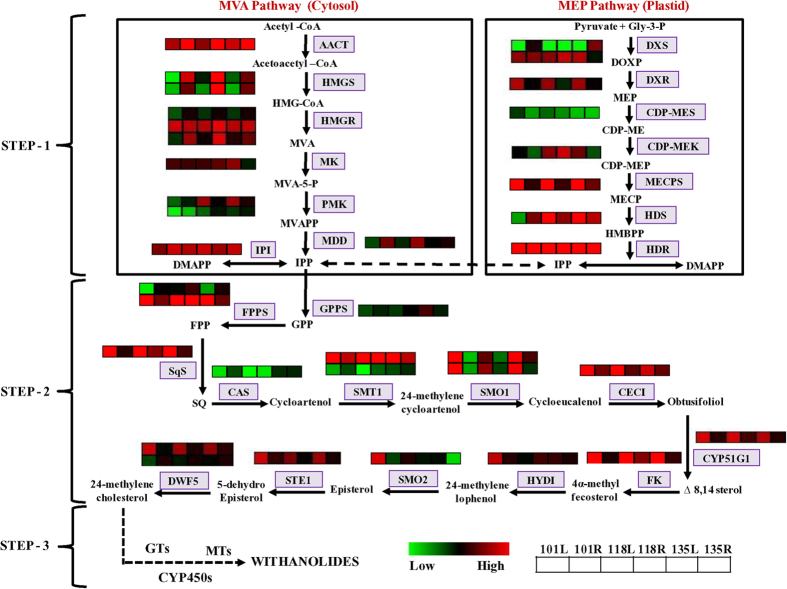
Triterpenoid biosynthesis and associated genes in *Withania*. Expression pattern of genes encoding enzymes involved in biosynthesis of IPP from MVA and MEP pathway (Step-1), 24-methylene cholesterol from IPP (Step-2) and involvement of putative gene families in biosynthesis of withanolides from 24-methylene cholesterol (Step-3). 101L, 118L, 135L, 101R, 118R, 135R in different heat maps represent abundance of contig in different chemotypes. Each row in heat map represents abundance of specific contig in different chemotypes.

Withanolides biosynthesis utilizes intermediates of terpenoid backbone^23^. Terpenoid backbone biosynthesis can be divided into two steps: Step 1 includes all the enzymatic steps involved in the biosynthesis of IPP via MVA and MEP pathway (Fig. 5). Both the pathway included seven enzyme-catalyzed reactions. Step 2 includes the biosynthesis of 24-methylene cholesterol, a central key molecule essential for biosynthesis of diversified withanolide moieties, from IPP (Fig. 5). BLASTx annotation suggests all the enzymes involved in various intermediate steps are encoded by the multiple copies of genes (Fig. 5). Each gene of MVA and MEP pathways as well as those involved in Step-2 was differentially expressed in six transcriptomes (Fig. 5). qRT-PCR analysis of the selected genes using NMITLI-101 chemotype validated the differential expression pattern observed in leaf and root tissues (Supplementary Fig. 3). In addition to leaf and root, flower and fruit tissues were also included in the analysis. Out of 12 genes, maximum expression of 7 genes (*MK, PMK, SQS, HYD*1, *DWF5a, DXS, DXR* and *CDPMK*) was observed in the fruit tissue (Supplementary Fig. 3). Transcript accumulation of *HMGR* was observed highest in root whereas *FPPS* expression was noted higher in leaf (Supplementary Fig. 3).

In most of the cases, more than one unigene was assigned to the same enzyme (Fig. 5). Such unigenes may represent different fragments of a single transcript, different members of a gene family, or both. Contigs obtained from combined assembly were used to analyze expression pattern of each gene because of their presence in both the tissues. For identification of alternatively spliced forms/multiple copies of unigenes encoding different enzymes catalyzing various enzymatic steps of the terpenoid backbone biosynthesis, *Withania* leaf assembled transcriptome data present in public domain^25^ was utilized (Supplementary Table S4). Maximum number of genes (contigs) were identified for *HMGR* (5 genes) followed by *FPPS* (4 genes) whereas for *CEC1* and *MECPS*, only partial sequences were observed (Supplementary Table S4). We observed alternative spliced variants for 8 genes in Step-1 (MVA and MEP pathway) and for 3 genes involved in Step-2 (Supplementary Table S4). All the genes with more than one number differ in their amino acid residues and molecular weight/pI (Supplementary Table S4).

### Involvement of *WsDWF5* in withanolide biosynthesis

Enzyme sterol ∆7-reductase is encoded by *DWF5* gene and catalyzes biosynthesis of 24-methylene cholesterol, a central precursor molecule for biosynthesis of variety of withanolides^13^,^14^. Our transcriptome analysis identified two genes encoding for DWF5 enzyme in *Withania. WsDWF5a* is highly expressed in leaf whereas *WsDWF5b* showed higher expression in root of three different chemotypes (Fig. 5a). We selected *WsDWF5a* for the Virus Induced Gene Silencing (VIGS) experiment to study its involvement in the withanolide biosynthesis.

**Figure 6:**
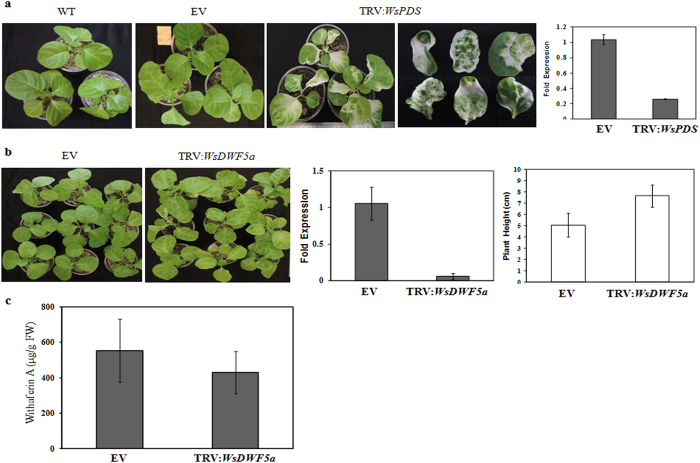
Virus induced gene silencing of *WsPDS* and *WsDWF5a* in *Withania*. (**a**) Phenotype of wild type (WT), TRV1 and TRV2 infected empty vector control (EV), TRV1 and TRV2:*WsPDS* infected (TRV:*WsPDS*) plants and reduced level of transcripts of *WsPDS* in *PDS* silenced plants. (**b**) Phenotype of EV and TRV1 and TRV2:*WsDWF5a* infected (TRV:*WsDWF5a*) plants, significantly decreased level of transcripts of *WsDWF5a* and increase in plant height in *DWF5a* silenced plants. (**c**) Reduced level of major withanolide in leaf (withaferin A) in *DWF5a* silenced plants as compared to EV.

Photobleaching detected in the newly emerged two weeks post infiltrated leaves of the *WsPDS* silenced plants (Fig. 6a) suggested effectiveness of TRV-based VIGS approach to promote gene silencing in *Withania*. qRT-PCR analysis of all the infiltrated plants showed significantly decreased *WsPDS* gene expression as compared to the control (Fig. 6a). Similarly, TRV2:*WsDWF5a* construct, harbouring 287 bp fragment of *WsDWF5a* (including 5-’UTR region) was used to silence the *DWF5a* gene. Plants generated through VIGS for *WsPDS* as well as *WsDWF5a* were analyzed for successful infection through expression of coat protein gene. In both cases, presence of amplicons suggests infection by virus (Supplementary Fig. 4). To test the successful silencing, infiltrated plants were analysed by PCR using a set of TRV1 and TRV2 specific primers. Leaves of control (EV), as well as TRV2:*WsDWF5a* plants displayed typical viral infection phenotype with an enhanced plant height (~51%) in TRV2:*WsDWF5a* plants. Significant reduction in the transcript level of *WsDWF5a* (~94%) was observed in TRV2:*WsDWF5a* plants as compared to the empty vector control plants (Fig. 6b). To determine the effect of *WsDWF5a* silencing on transcript accumulation of other genes (*HMGR, DXS, SQS, CAS, CYP51G*1, *HYD*1, *STE*1 and *DWF5*b) involved in intermediate steps of withanolide biosynthesis pathway, qRT-PCR analysis was carried out (Supplementary Fig. 5). Expression of most of the genes (*HMGR, DXS, SQS, CAS, CYP51G*1 and *STE*1) was significantly reduced in TRV2:*WsDWF5a* plants. Maximum reduction in expression was observed in *STE*1, an immediate upstream gene to *DWF5*. Interestingly, enhanced expression of *WsHYD*1 was observed in *WsDWF5a* silenced plants as compared to control (EV) plants. In case of *WsDWF5b*, a paralog of *WsDWF5a,* no significant change in transcript levels was observed which indicates minimal possibilities of off-target silencing (Supplementary Fig. 5). Silencing of *WsDWF5a* also affected the level of main withanolide content in leaf tissues of *Withania*. Content of withaferin A was reduced by ~22% in *WsDWF5a* silenced plants as compared to the control (EV) plants (Fig. 6C; Supplementary Fig. 6).

### Gene families involves in withanolide biosynthesis

It is assumed that using 24-methylene cholesterol as substrate, various secondary transformations including oxidation/reduction, hydroxylation, glycosylation and methylation takes place to synthesize tissue-specific as well as chemotype-specific withanolides. It is assumed that putative candidate gene families involved in secondary transformations (Step-3, Fig. 5) might be cytochrome P450s (CYP450s), glycosyltransferases (GTs), methyltransferases (MTs) and transcription factors (TFs), which may provide tissue-specific biosynthesis and\or accumulation of withanolides. Annotated contigs from the combined assembly were used for the identification of members of these gene families using name search. Out of total members identified from these gene families, differentially expressed members were identified to predict their putative involvement in the specific withanolide biosynthesis (Supplementary Table S5).

**Figure 7:**
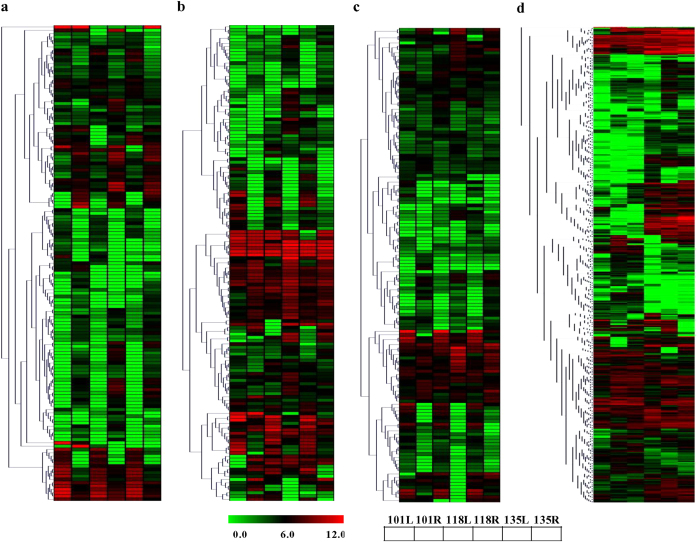
Identification and differential gene expression of members of gene families putatively involved in withanolide biosynthesis. Members of cytochrome P450 (**a**), glycosyltransferase (**b**), methyltransferase (**c**) and transcription factor (**d**) gene families. Each row of heat map represents expression pattern of different contig. Each column in heat maps represent expression in leaf (L) and root (R) tissues of different chemotypes (NMITLI-101; NMITLI-118 and NMITLI-135).

Out of all the contigs, 183, 120 and 224 unigenes are annotated as members of CYP450 gene family against TAIR10, NR and tomato genome databases, respectively (Supplementary Table S5). On the basis of annotation results and excluding redundant contigs from different databases, a total of 228 unigenes are identified as members of the CYP450 gene family from *Withania*. Such a large family of CYP450 has also been reported in other plants such as *Panax ginseng*^26^. Though highest number of CYPs was annotated using tomato genome database, a large number (100 unigenes) were commonly annotated from three databases (Supplementary Fig. 7a). Expression analysis suggested that out of 228 unigenes related to CYP450s, 143 unigenes are differential expressed in leaf and root tissues of three different chemotypes (Fig. 7a). Some of these might be involved in chemo- or tissue–specific biosynthesis of specific-withanolides.

Withanosides or siotoindisides are glycosylated steroidal lactones having one or more glucose units attached to C-3 or C-27 positions and might be synthesized by the action of GTs 27. On the basis of annotation results and excluding redundant contigs from different databases, a total of 259 unigenes are identified as members of GT gene family (Supplementary Table S5). Maximum number of unigenes was annotated as members of GT contigs using *Arabidopsis* information resource. A very small number, 5 unigenes, were commonly annotated as GTs from three databases (Supplementary Fig. 7b). Interestingly, a large number of unigenes (144) was annotated as GTs showed differential expression pattern in different chemotypes as shown in Fig. 6b. Out of 259 GTs indentified from transcriptome data, deduced amino acid sequence of 20 members possess plant secondary product glycosyltransferases (PSPG) box^28^,^29^ at their C- terminal end (Supplementary Fig. 7c).

A total of 419 unigenes were identified as members of MT gene family (Supplementary Table S5). Out of total unigenes as members of MT gene family, 106 were commonly annotated through three different databases (Supplementary Fig. 7d). Maximum annotation (279 members) was observed against the tomato genome database as well as 90 members were uniquely annotated through tomato genome database (Supplementary Fig. 7d). Out of total MTs, 143 unigenes showed differential expression in different chemotypes (Supplementary Table S5; Fig. 7c).

Various studies suggest that secondary plant product biosynthesis is regulated by transcription factors^30^,^31^. Some of these transcription factors have been used to enhance biosynthesis of specific molecules in homologous and heterologous systems^32^,^33^,^34^. We hypothesized that modulated expression of *Withania* transcription factors might be involved in biosynthesis of specific withanolides. To study this, search for members of different transcription factor gene families was carried out in annotated transcriptomes. A total of 1210 unigenes were identified as the members of different transcription factor gene family (Supplementary Table S5). Out of 1210 unigenes, 624 unigenes were differentially expressed (Fig. 7d). Out of total members of different transcription factor gene families, maximum annotation (599 members) was observed against tomato genome database and 68 members were commonly annotated through three different databases (Supplementary Fig. 7e).

### Identification of SSRs

Simple sequence repeats (SSRs) are used in genetic breeding applications of plants and are highly abundant in the 454 transcriptome sequences. NGS technologies have been used for development of molecular markers in the non-model organisms^35^,^36^,^37^. Total contigs and singletons from leaf tissue of three different chemotypes were searched for SSR identification. In NMITLI-101 chemotype, 683 and 653 contigs were observed to contain 729 and 703 SSRs from leaf and root tissues, respectively^23^. Similarly, 1684 and 2283 singletons containing 1824 and 2259 SSR sequences from leaf and root tissues respectively were identified from NMITLI-101 chemotype^23^.

In NMITLI-118 chemotype, a total of 514 and 1015 SSR sequences were identified in 478 contigs and 939 singletons from leaf tissues and 581 contigs and 1193 singletons containing 636 and 1267 SSR sequences were identified from root tissues (Supplementary Table S6 and S7). Most abundant SSRs in contigs of NMITLI-118 leaf (320 SSRs) and roots (394 SSRs) were tri-nucleotide repeats. In singletons from leaf and root tissue of NMITLI-118, most abundant SSRs were di-nucleotide repeats (Supplementary Table S7).

A total of 443 and 384 contigs were found to possess 470 and 414 SSRs in leaf and root tissues, respectively of NMITLI-135 chemotype (Supplementary Table S6 and S7). Tri-nucleotides were the most abundant SSRs in contigs as well as singletons of leaf and root tissues (Supplementary Table S6 and S7). In contigs of leaf and root tissues, penta-nucleotide repeats were found to be absent and tetra-nucleotide repeats were the least (4 and 3 in leaf and root tissue respectively). Identification of these markers and further study to associate them with differentially expressed genes might help in developing markers for chemotypes related to specific withanolides.

## Discussion

Transcriptome sequencing is a cost effective method for developing genomic information from different non-model medicinal plants for which no reference genome is available. The transcriptome data, generated in this study, is highly valuable to shed light on the withanolide biosynthesis pathway, gene families involved in specific withanolide biosynthesis, chemodiversity, paralogs of genes involved in terpenoid biosynthesis pathway as well as molecular marker studies. Annotation of different transcriptomes suggested that more than 40% of unigenes are efficiently matched with the genomic/transcriptomic resources present in the public domains or databases. Analysis also suggests that unigenes and singletons, developed in this study, have higher percentage of annotation against tomato genome database as compared to other databases (Supplementary Fig. 1). Higher percentage of similarity and synteny with tomato as compared to other plants may be due to both, tomato and *Withania*, being the members of *Solanaceae* family.

Functional GO assignments represented a large number of the diverse GO terms to unigenes which highlights the diversity of genes represented in *Withania* transcriptome data (Fig. 3). Highest percentage of genes were grouped in ‘other metabolic process’ which may allow for the identification of novel genes involved in the secondary metabolite biosynthesis pathways from *W. somnifera*. Mapping of unigenes onto the KEGG pathways (Table 1) helped in identification of large number of unigenes involved in biosynthesis of various secondary metabolites in *Withania. Withania* possesses ginseng like properties and similar functional annotation of *P. ginseng* was carried out by using Gene Ontology (GO) classifications, KEGG orthology and structural domain database. Through these analyses, isoprenoid and putative ginsenoside pathway genes were identified in *P. ginseng*^38^.

Differential gene expression analysis (Fig. 4) suggests that a large number of genes are differentially expressed in leaf and root tissues of three distinct chemotypes of *Withania*. Among all 12 clusters, genes grouped in clusters 9 and 12 expressed highly in leaf tissues as compared to roots and might be involved in withaferin A biosynthesis. Similarly, some of the genes with higher expression in roots as compared to leaves (in cluster 8) might be associated with withanolide A biosynthesis.

Surprisingly, for each enzymatic step of triterpenoid backbone biosynthesis, more than one contig was identified through annotation of assembled sequences. This suggested that these contigs might be members of the multigene families or part of same gene without overlapping sequences to form a single large contig. To get more insight on this aspect, all the contigs related to terpenoid backbone biosynthesis pathway (up to 24-methylene cholesterol) were mapped on to the assembled data of *Withania* leaf transcriptome (developed using Illumina sequencing platform) present in public domain to get full-length sequence of each contig. This analysis shed light over identification of paralogs for most of the genes encoding enzymes catalyzing different steps. Maximum numbers (5 genes) of paralogs were observed for *WsHMGR* suggested that this gene might have undergone various gene duplication events. Similarly, in many other plant species which produce more or wider variety of terpene compounds such as *Gossypium raimondii* (9 HMGR genes), *Glycine max* (8 HMGR gene), *Zea mays* (7 HMGR genes) and *Populus trichocarpa* (6 HMGR genes), a large number of HMGR genes have been identified^39^. In most of the genes, difference in their encoded amino acid sequences has been due to the presence of single nucleotide polymorphism. As ploidy level of *Withania* genome is still unexplored, it is hard to predict presence of paralogous genes. A model plant, *Arabidopsis thaliana* has undergone at least three rounds of polyploidy and many paralous genes display different expression pattern^40^,^41^.

Biosynthesis of withanolides, the signature secondary metabolites as well as active constituents of *W. somnifera,* takes place from triterpenoid pathway through metabolic divergence at the level of 24-methylene cholesterol^42^. In our study, silencing of *WsDWF5a* gene encoding enzyme which catalyzes biosynthesis of 24-methylene cholesterol caused significant reduction in level of withaferin A biosynthesis. This suggested role of 24-methylene cholesterol as a key precursor molecule for withanolides biosynthesis. Down-regulated expression of upstream pathway genes, except *HYD*1, in silenced *WsDWF5a* plants suggested reduction in substrate flux due to accumulation/sufficient availability of the intermediate compounds. Enhanced expression of *WsHYD1* suggested possible involvement of HYD1 in synthesis of of plant sterols other than 24-methylene cholesterol. Increase in plant height of *WsDWFa* silenced plants suggest possible regulatory role of withanolides in plant growth and development processes.

Further steps of the biosynthetic pathway downstream to 24-methylene cholesterol for specific withanolide biosynthesis are still unresolved. Therefore, an attempt to identify major gene families involved in the secondary conversion steps of withanolide biosynthesis has been made (Supplementary Table S5). Identification of large number of differentially expressed CYP450s indicates involvement of some of these CYP450s in chemodiversity as well as candidate genes for specific withanolide biosynthesis in tissue specific manner. Glycosyltransferases are the key enzymes involved in biosynthesis of sitoindosides in *Withania* and utilize a broad range of substrates such as sterols and withanolides^43^. Identification of GTs possessing PSPG box suggested their putative role in biosynthesis of secondary plant products in *Withania*.

Identification of SSRs in leaf and root tissue of three different chemotypes (Supplementary Table S6 and S7) will help in the dissection of complex genetic background of *Withania* especially in relation to distinct chemotypes as well as marker assisted breeding in future. Information gathered, in this study, will help to get more insight about *Withania* research and develop strategies for enhanced biosynthesis of specific withanolide in homologous or heterologous systems. Overall, this study generates a huge, valuable genomic resource to explore chemotype- as well as tissue-specific withanolide biosynthesis and single nucleotide polymorphism based chemodiversity in future.

## Methods

### Plant material

Chemotypes of *W. somnifera* (NMITLI-101, NMITLI-118, and NMITLI-135) were developed under the CSIR-New Millennium Indian Technology Leadership Initiative (CSIR-NMITLI) Program^16^ and were grown at experimental plot in the institute under standard cultivation techniques. Young leaves (3^rd^-5^th^ leaf from top) from one year old field grown plants were collected, washed with sterile water, frozen in liquid nitrogen and stored in −70 °C until use. Each chemotype was uprooted and main root (avoiding lateral roots) was excised with the help of sterile blade, washed with sterile water, frozen in liquid nitrogen and stored in −70 °C until use.

### Phytochemical analysis

Extraction and analysis of withanolides from leaf and root tissues (4 g) of different chemotypes was carried out essentially according to Chaurasiya *et al.* (2009)^16^. The chloroform fractions were pooled, concentrated to dry powder, dissolved in HPLC grade methanol, filtered (Millex GV; 13 mm, 0.22 μm filters) and subjected to HPLC according to method described by Gupta *et al.* (2011)^13^. The content of the specific withanolides in each sample was calculated using calibration curves (peak area versus amount of the marker withanolides). The average content of the withanolides of at least three independent samples of each tissue was calculated and expressed as percentage of dry weight of the tissue. Data are expressed as mean ± standard deviation (SD) of all replicates.

### Library construction, transcriptome sequencing and analysis

For isolation of total RNA, frozen tissues (leaf and root) of chemotypes were ground to a fine powder in liquid nitrogen and total RNA was extracted using Spectrum Plant Total RNA Kit (Sigma–Aldrich, USA). Preparation of double-stranded (ds) cDNA and library preparation for sequencing was carried out according to Gupta *et al.* (2013)^23^. The sequencing was performed using half plate run for each sample on 454-GS FLX sequencing platform (454 Life Sciences, Roche, USA) using GS FLX Titanium Kit.

The 454 raw read sequences were filtered for weak signals, low quality sequences and trimmed for 454 adaptor sequences to yield high quality (HQ) sequences (99.5% accuracy on single base reads). The data from the 454 read sequences were *de novo* assembled into unique sequences using Roche GSAssembler (version2.5.3). For combined assembly, assembled read sequences from leaf and root libraries of different chemotypes (NMITLI-101, NMITLI-118 and NMITLI-135) were tagged, pooled and assembled together to generate contigs of larger size with 40 base pair overlap and 95% identity. The contigs and singletons of individual leaf and root libraries as well as from combined assembly were annotated using standalone version of BLASTx program against *Arabidopsis* protein database at The Arabidopsis Information Resource (TAIR; http://www.arabidopsis.org; TAIR10), the NCBI non redundant protein (NR) database (http://www.ncbi.nlm.nih.gov), and tomato (http://solgenomics.net/ organism/Solanum_lycopersicum/genome; version ITAG2.3) with an E-value cut-off of 10^−5^ and extracting only the top hit for each sequence. Contigs generated from combined assembly were also annotated using standalone version of BLASTx program against potato (http://solgenomics.net/organism/Solanumtuberosum/ genome;v3.4) and NCBI-CDD (Conserve Domain Database) with an E-value cut-off of 10^−5^. Synteny of *Withania* transcripts as well as tomato transcripts with 13 chromosomes of tomato was plotted using Circos software package^44^.

### Functional annotation and classification of assembled transcriptomes

To assign function to each unigene generated from combined assembly, gene ontology (GO) analysis was performed using GO annotation in online TAIR Database (http://www.arabidopsis.org/). Unigenes were classified under the categories of Cellular Component, Molecular Function and Biological Process. The TAIR IDs of all the unigenes (contigs and singletons) were retrieved from TAIR10 annotation^45^.

The assignments of polypeptides encoded by unigenes generated from combined assembly were mapped into various metabolic pathways according to the Kyoto Encyclopedia of Genes and Genomes (KEGG)^46^. Enzyme commission (EC) numbers were assigned to unique sequences, based on the BLASTx search of protein databases, using an E-value cut-off of 10^−5^.

### Digital differential gene expression analysis

Differential expression of genes was calculated by using R value^47^. Genes with R value ≥12 were considered as differentially expressed. Hierarchical clustering (HCl) of log-transformed expression data was carried out using Euclidean Distance Matrix and Average linkage clustering method^48^. All identified differentially expressed genes were divided into 12 different clusters according to their expression pattern in leaf and root tissues of different chemotypes.

### Identification of contigs related to terpenoid biosynthesis and expression analysis

On the basis of annotation results of contigs, genes involved in the MEP and MVA pathways as well as for the triterponoid backbone biosynthesis were identified. To identify the contig related to each enzymatic step, gene or enzyme names were used for the search in annotation results. Full-length sequence of genes encoding enzymes catalyzing different step of the MVA and MEP pathway (up to 24-methylene cholesterol) were obtained using assembled data of *Withania* leaf transcriptome (SRR1015957- GBHJ01000001-GBHJ01073511) generated through illumina sequencing platform^25^. Assembled data of identified contigs were again translated to proteins and their homology in translated region was calculated manually on the basis of genes or enzymes characterized from different plants. To study digital gene expression among leaf and root tissues of different chemotypes of *Withania*, the log2 converted values, as representation of reads to form each contig associated with biosynthesis of terpenoid back bone, were subjected in MeV (version 4.8.1) for construction of heat maps and clusters.

### Identification of members of gene families involved in specific withanolide biosynthesis

Annotation results of unigenes generated from combined assembly of *Withania* were used for identification of members of CYP450, GT,, MT and transcription factor gene families. Sequences for contig of each member of different gene family were retrieved from combined assembly. All the members of gene families identified were analyzed manually and validated using BLAST search using NCBI database. Vein diagrams were plotted using online tools (http://bioinfogp.cnb.csic.es/tools/venny/;http://bioinformatics.psb.ugent.be/webtools/Venn/). Heat maps were generated through MeV (version 4.8.1) by using log2 converted transcripts per million (tpm) values for each contig and Hierarchical clustering (HCl) of log-transformed expression data was carried out using Euclidean Distance Matrix and Average linkage clustering method^48^.

### VIGS construct preparation, transformation and infiltration

Silencing of *DWF5a* gene through VIGS in *Withania* was employed using standard protocol established by Singh *et al.* (2015)^49^. *WsDWF5a* gene fragment (287 bp) was amplified using cDNA as a template and gene-specific primers. This region includes 5-UTR as well as part of coding region and has very less homology with *WsDWF5b* (Supplementary Fig. 8). Gene fragment was cloned into pTRV2 vector at EcoR1 and Sac1 site to form pTRV2:*WsDWF5a* construct. Sequences of oligonucleotides used for cDNA amplification and construct preparation are mentioned in Supplementary Table S8. For silencing of *WsPDS*, TRV2:*WsPDS* construct prepared by Singh *et al.* (2015)^49^ was used in this study.

For transformation, both the pTRV1 and pTRV2 derivatives were introduced into *Agrobacterium tumefaciens* strain LBA4404 through electroporation. *Agrobacterium* cultures were grown as described by Liu *et al.* (2002)^50^.Cultures were centrifuged at 3,000× g for 10 min at 4 °C and cells were resuspended in infiltration medium (10 mM MES, 200 μM acetosyringone, 10 mM MgCl2) for incubation at 28 °C for 3 h. pTRV1 was co-infiltrated with pTRV2:*WsPDS,* pTRV2:*WsDWF5a* or pTRV2 (EV) in 1:1 ratio on the abaxial surface of leaves in 4-leaf-staged *Withania* plants. After infiltration, plants were kept in dark for overnight and then placed in glass house under controlled growth conditions (22 °C and 16h day/8h night cycle). Newly emerged leaves from infiltrated plants showing typical viral infection symptoms were collected at 30 days post infiltration (dpi) and stored at −80 °C for further analysis. At least 15 independent plants were used for infiltration experiment for each construct and 6 independent infiltrated plants were used for further analysis.

### Quantitative gene expression analysis

Quantitative expression of genes in different tissues of a chemotypes was analyzed using Real-Time PCR Detection System and Fast SYBR Green PCR Master Mix (ABI 7500, Applied Biosystems, USA). For each primer set, a control reaction was also included having no template. Actin gene from *W. somnifera* was used as internal control to estimate the relative transcript level of the genes analyzed. Data from qRT-PCR amplification was analyzed using comparative Ct (2^−ΔΔct^) method^51^,^52^. Fold change in expression was calculated as 2^-ΔΔct^ using ΔCt values. All the experiments were repeated using three biological replicates and the data were analyzed statistically (±Standard Deviation). Gene specific oligonucleotides used for qRT-PCR analysis are provided in Supplementary Table S8.

### Identification of simple sequence repeats (SSRs)

For identification of SSR motifs, contigs and singletons generated from transcriptome sequence of leaf and root tissues of three different chemotypes were used in a Microsatellite program (MISA) (http://pgrc.ipk-gatersleben.de/misa/ misa.html). Analysis included identification of microsatellites from di-nucleotide to hexa-nucleotides. The parameters used for simple sequence repeats (SSRs) were at least 6 repeats for di- and 5 for tri-, tetra-, penta- and hexa- nucleotide. Both perfect (i.e. contain a single repeat motif like such as ‘ATC’) and compound repeats (i.e. composed of two or more motifs separated by 100 bases) were identified by the analysis. Mono-nucleotide repeat motifs were not considered in the analysis due to chances of homopolymer tailing in ESTs generated using 454 pyrosequencing.

## Additional Information

**How to cite this article**: Gupta, P. *et al.* Comparative transcriptome analysis of different chemotypes elucidates withanolide biosynthesis pathway from medicinal plant *Withania somnifera. Sci. Rep.* **5**, 18611; doi: 10.1038/srep18611 (2015).

## Supplementary Material

Supplementary Information

## Figures and Tables

**Table 1:** Unigenes related to secondary metabolite biosynthesis in *Withania somnifera.*

KEGG Pathways	Contigs	Singletons
Anthocyanin biosynthesis [PATH:00942]	2	3
Brassinosteroid biosynthesis [PATH:00905]	10	19
Caffeine metabolism [PATH:00232]	6	4
Carotenoid biosynthesis [PATH:00906]	61	26
Cutin, suberin and wax biosynthesis [PATH:00073]	10	11
Diterpenoid biosynthesis [PATH:00904]	16	17
Flavone and flavonol biosynthesis [PATH:00944]	11	4
Flavonoid biosynthesis [PATH:00941]	32	20
Indole alkaloid biosynthesis [PATH:00901]	2	0
Isoquinoline alkaloid biosynthesis [PATH:00950]	15	21
Limonine and pinene degradation [PATH:00903]	44	59
Monoterpenoid biosynthesis [PATH:00902]	9	7
Nicotinate and nicotinamide metabolism [PATH:00760]	22	13
Phenylpropanoid metabolism [PATH:00940]	128	97
Sesquiterpenoid and triterpenoid biosynthesis [PATH:00909]	17	14
Steroid biosynthesis [PATH:000100]	34	32
Stibenoid diarylhepatanoid and gingerol biosynthesis [PATH:00945]	59	52
Terpenoid backbone biosynthesis [PATH:00900]	62	40
Tropane, piperidine and pyridine alkaloid biosynthesis [PATH:00960]	19	25
Ubiquinone and other terpenoid-quinone biosynthesis [PATH:00130]	38	26
Zeatin biosynthesis [PATH:00908]	12	13
Total	609	503
