# Supplementary material for: Comparative transcriptome analysis of different chemotypes elucidates withanolide biosynthesis pathway from medicinal plant Withania somnifera
Source: Sci Rep. 2015 Dec 21;5:18611. doi: 10.1038/srep18611 (PMC4685652; doi:10.1038/srep18611)
Supplement: Supplementary Information [file srep18611-s1.pdf]

Supplementary Information

Comparative transcriptome analysis of different chemotypes elucidates withanolide biosynthesis pathway from medicinal plant *Withania somnifera*

Parul Gupta[1], Ridhi Goel[1,3], Aditya Vikram Agarwal[1], Mehar Hasan Asif[1,3], Neelam Singh Sangwan[2], Rajender Singh Sangwan[2], Prabodh Kumar Trivedi[1,3,]*

[1]CSIR-National Botanical Research Institute (CSIR-NBRI), Rana Pratap Marg, Lucknow-226 001, INDIA

[2]CSIR-Central Institute of Medicinal and Aromatic Plants (CSIR-CIMAP), Lucknow-226015, INDIA

[3]Academy of Scientific and Innovative Research (AcSIR), Anusandhan Bhawan, 2 Rafi Marg, NewDelhi-110001, INDIA

***** Author for correspondence

E-mail address: prabodht@hotmail.com; prabodht@nbri.res.in

Tel: 91-522-2297958; **Fax:** 91-522-2205836, 2205839

Running Title: Chemotype-specific *Withania* transcriptomes

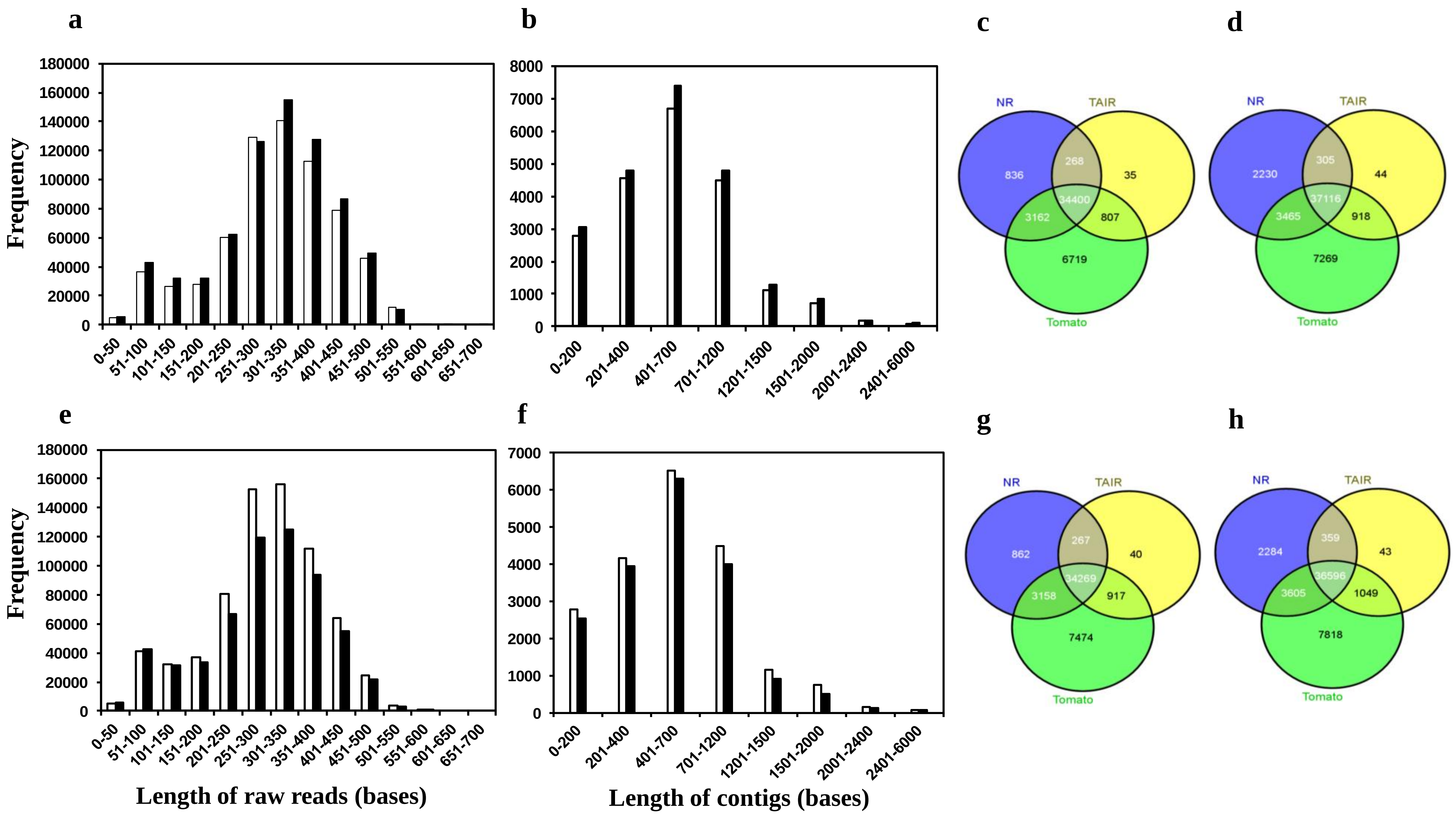

Supplementary Figure 1: Size distribution and annotation summary of contigs of NMITLI-118 and NMITLI-135. **(a)** HQ reads of NMITLI-118, **(b)** assembled contigs of NMITLI-118. Annotation of NMITLI-118 contigs against different databases **(c)** Leaf, **(d)** Root. **(e)** HQ reads of NMITLI-135, **(f)** assembled contigs of NMITLI-135. Annotation of NMITLI-135 contigs against different databases **(g)** Leaf, **(h)** Root. White and black bars represent reads and contigs from leaf and root transcriptome data respectively.

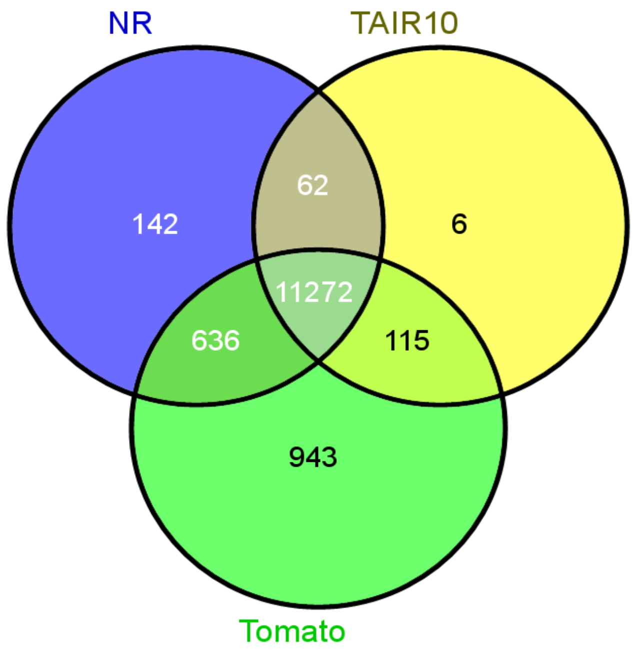

Supplementary Figure 2: Distribution of differentially expressed contigs annotated against TAIR10, NR and tomato genome database.

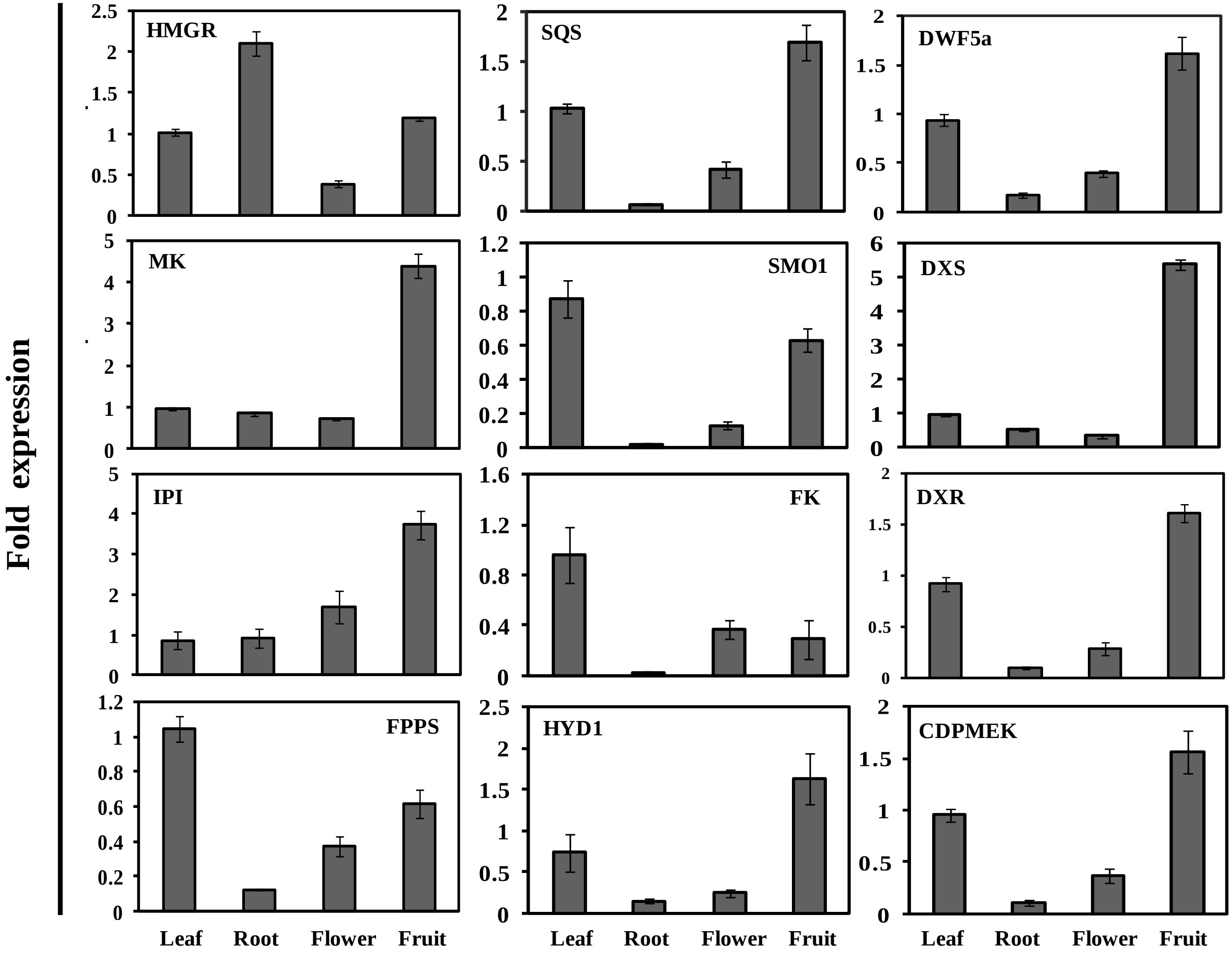

Supplementary Figure 3: Differential expression of selected genes involved in withanolide biosynthesis pathway in leaf, root, flower and fruit (green berries) tissues of NMITLI-101 chemotype. Expression of genes was measured through Real-time quantitative PCR analysis and is relative to leaf tissue. HMGR: 3-hydroxy-3-methylglutaryl-CoA reductase; MK: mevalonate kinase; IPI: isopentenyl diphosphate isomerase; FPPS: farnesyl diphosphate synthase; SQS: squalene synthase; SMO1: sterol-4α-methyl oxidase 1; FK: D14-sterol reductase; HYD1:; DWF5a: sterol Δ7 reductase; DXS: 1-deoxy-D-xylulose5-phosphate synthase; DXR: 1-deoxy-D-xylulose5-phosphate reductase; CDPMEK: 4-diphosphocytidyl-2-C-methyl-D-erythritol kinase.

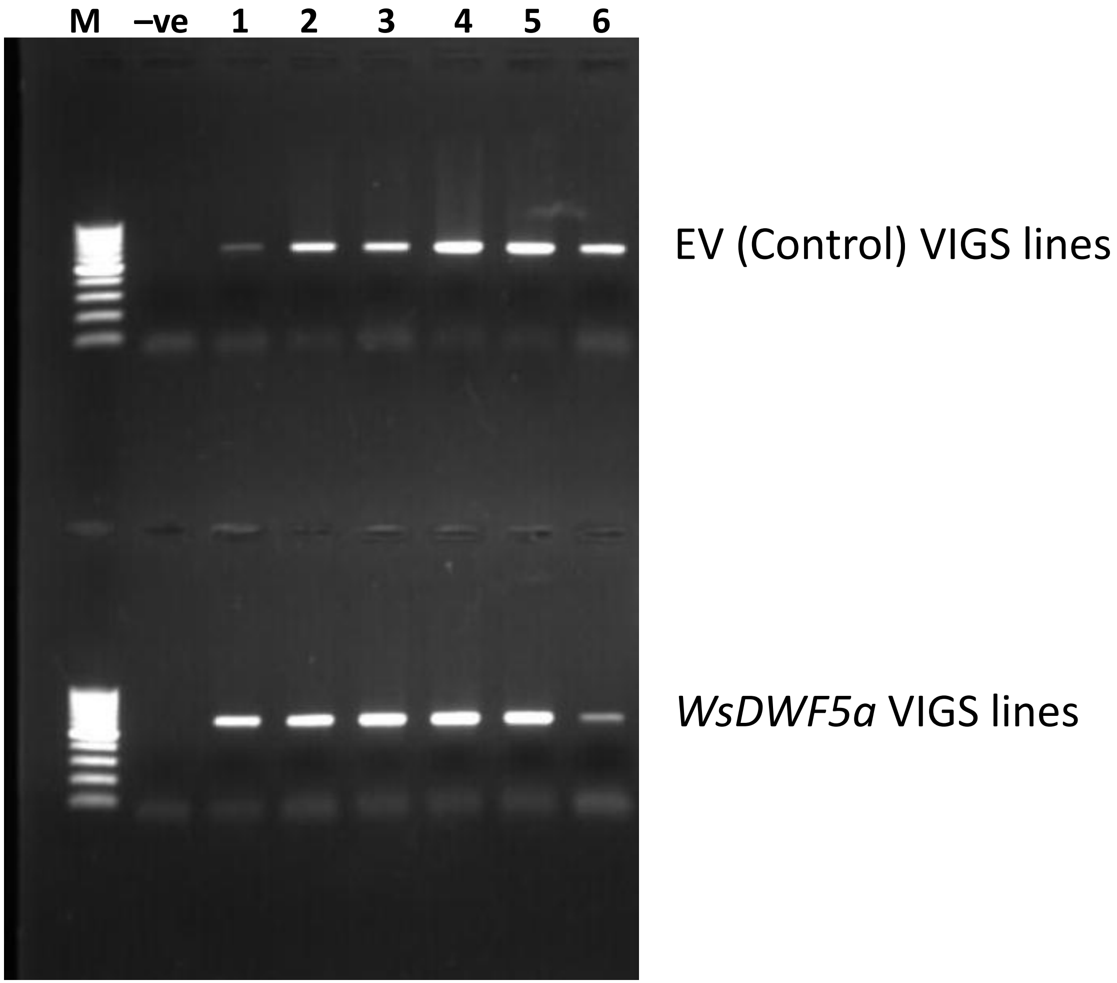

Supplementary Figure 4: Confirmation of virus infection in different VIGS lines. Expression of coat protein gene was analyzed in EV and WsDWF5a VIGS lines through semi-quantitative analysis using coat protein specific primers. Upper and lower panels show expression of coat protein genes in EV and *WsDWF5a* infected plants respectively. M, 100 bp ladder; -ve, negative controls; 1-6, different VIGS silenced lines.

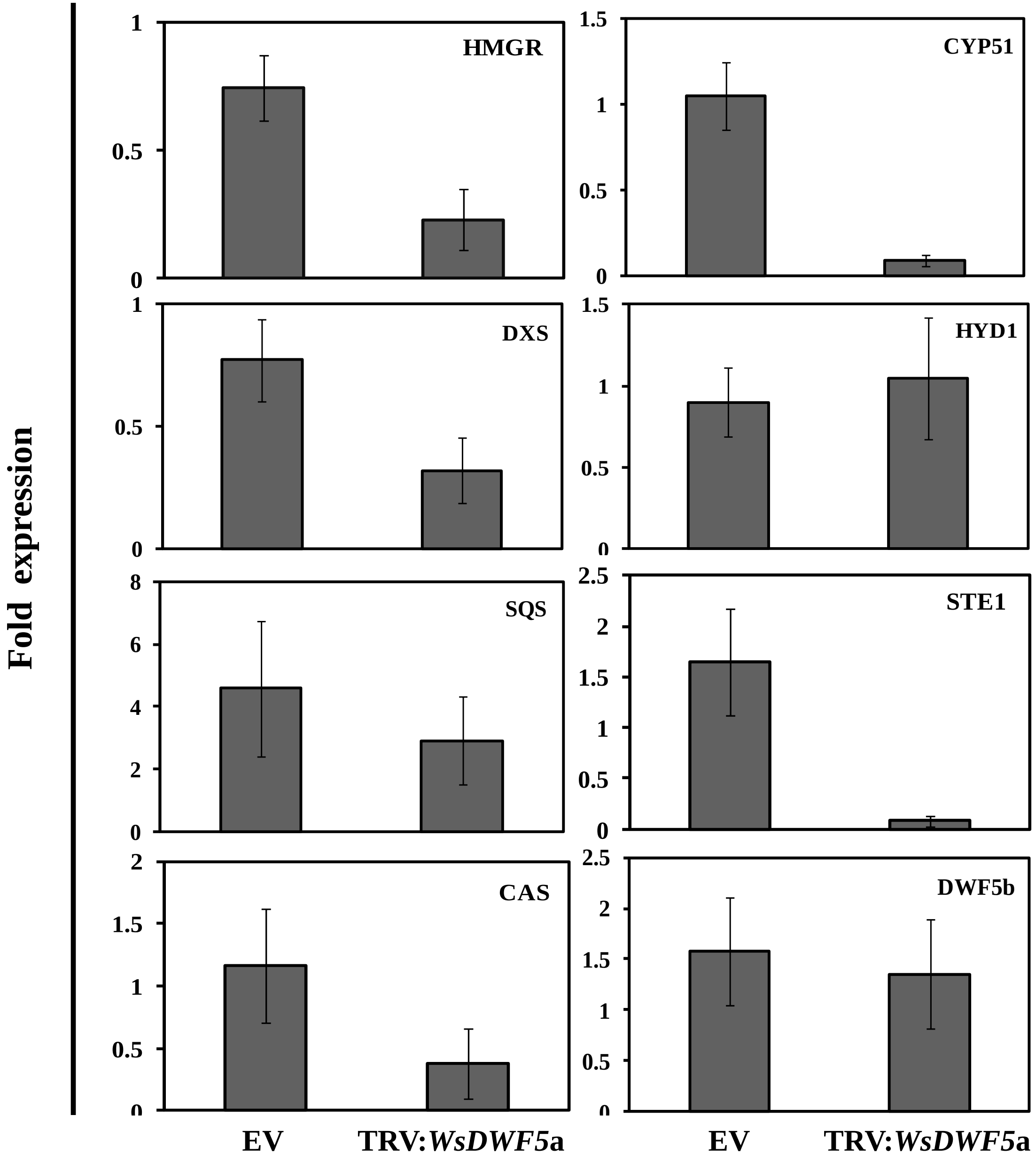

Supplementary Figure 5: Transcript accumulation of withanolide biosynthesis pathway genes in TRV1 and TRV2 infected control (EV) plants and TRV1 and TRV2:*WsDWF5a* containing *WsDWF5a* silenced plants.

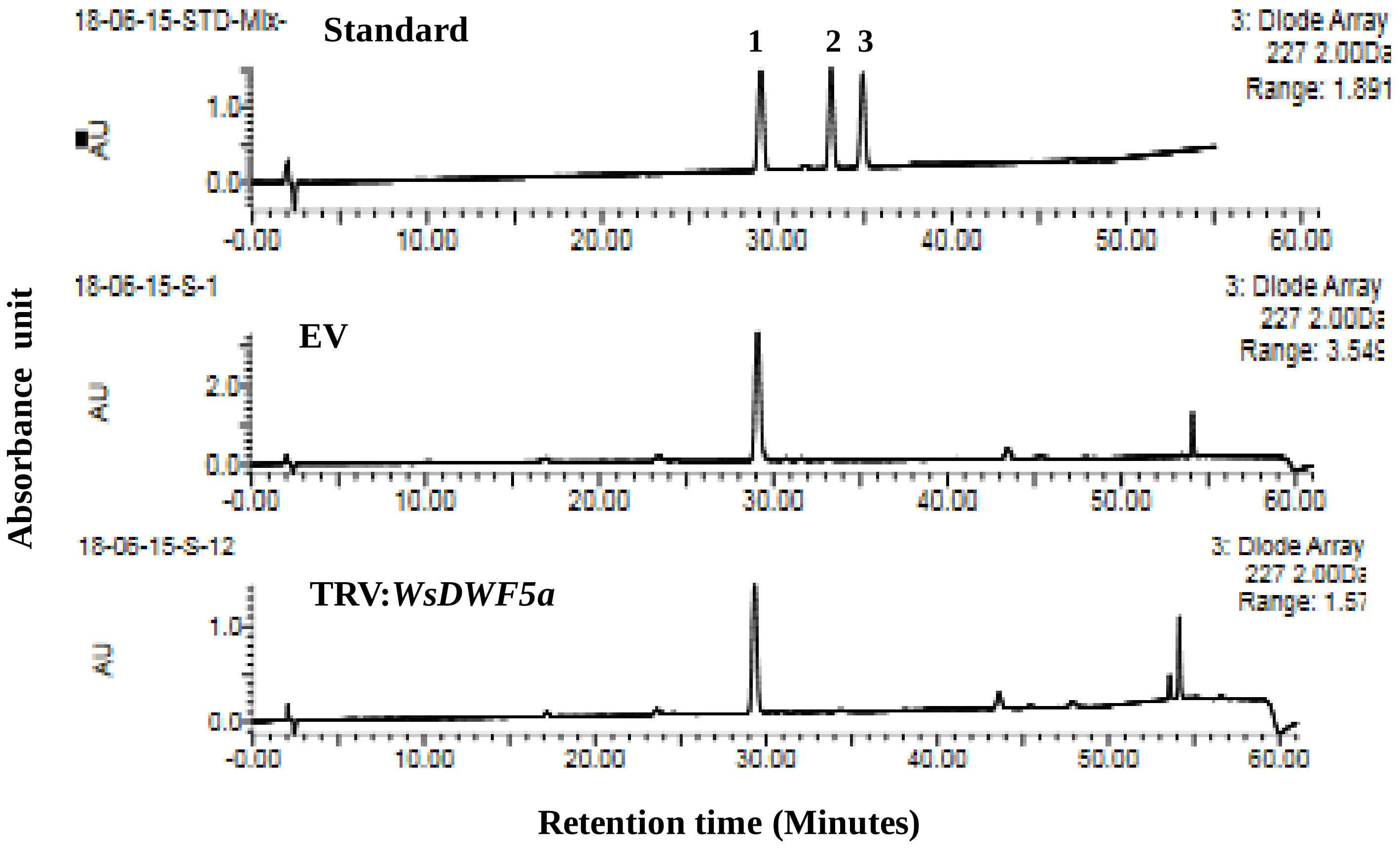

Supplementary Figure 6: HPLC profile of TRV1 and TRV2 infected control (EV) plants, TRV1 and TRV2:*WsDWF5a* containing *WsDWF5a* silenced plants and marker (standard) withanolide, (1: withaferin A; 2: withanolide A; 3: withanone).

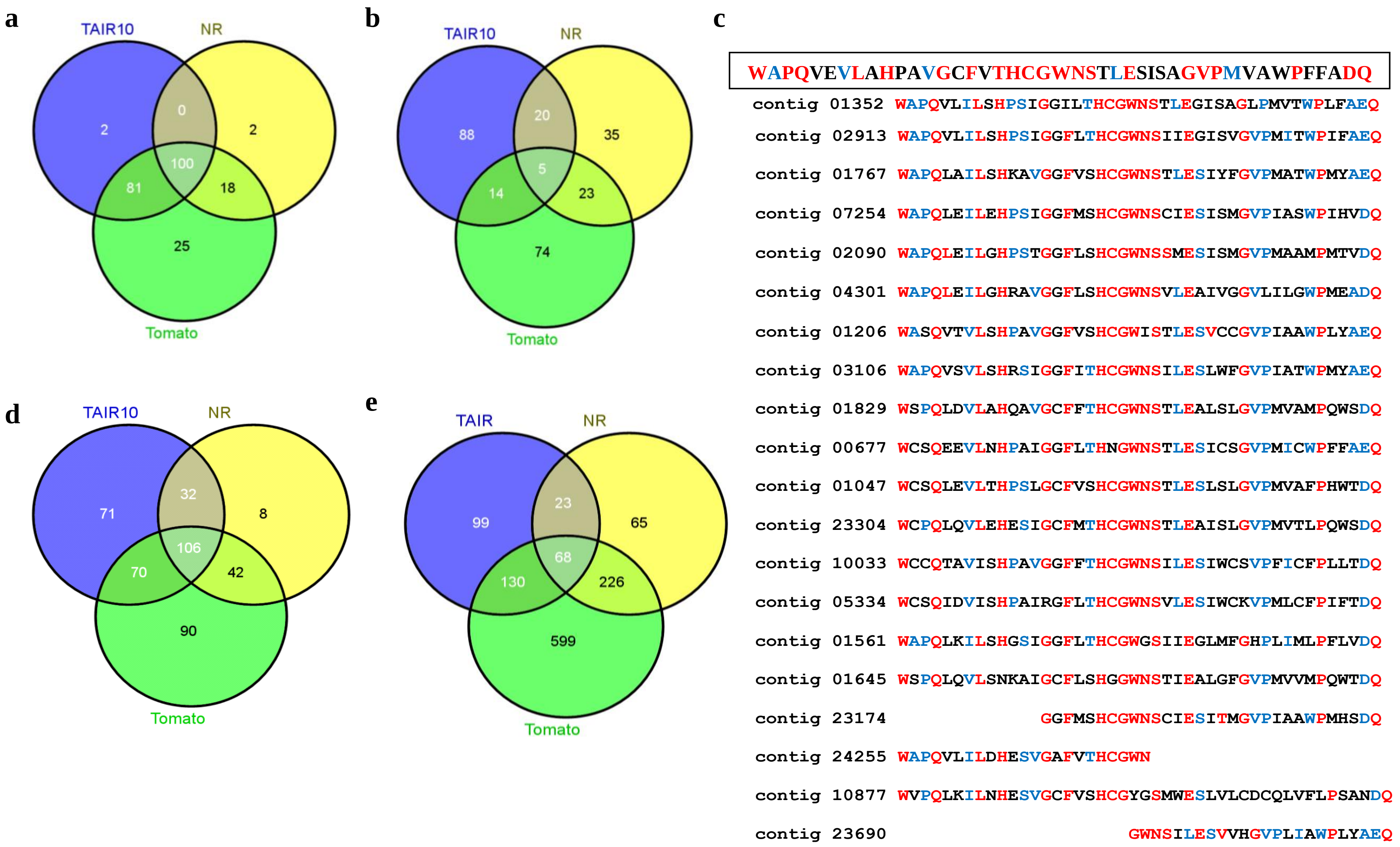

Supplementary Figure 7: Annotation of members of gene families putatively involved withanolide biosynthesis against different databases, **(a)** Cytochrome P450s, **(b)** Glycosyltransferases, **(c)** conserved sequence of PSPG box (in box) and members of glycosyltransferase gene family containing PSPG box in *Withania* transcriptome, **(d)** Methyltransferase and, **(e)** Transcription factors.

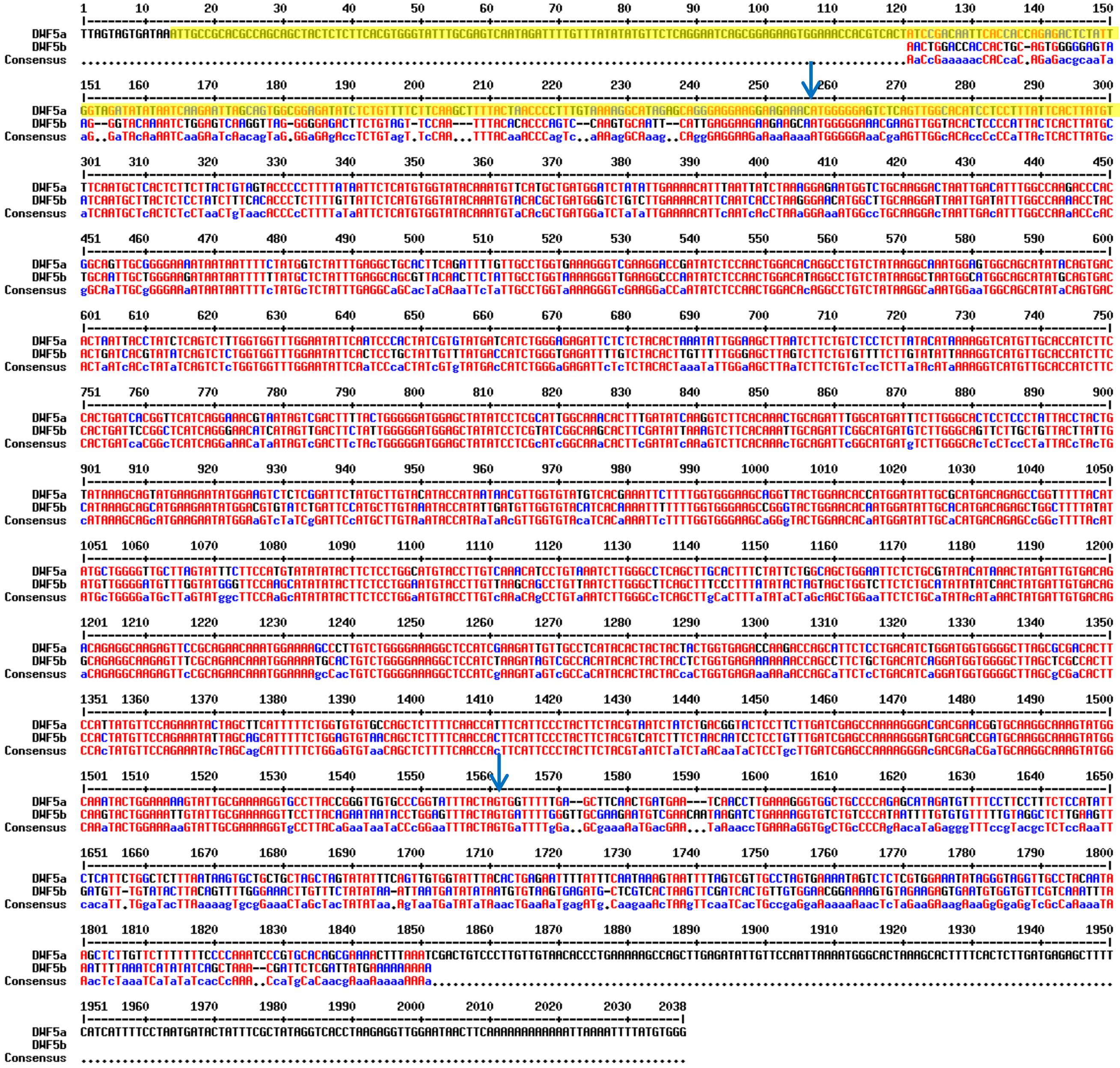

Supplementary Figure 8: Nucleotide sequence alignment of *WsDWF5a* and *WsDWF5b*. Translation start (ATG) and stop (TAG) codons are marked by the arrows. Nucleotide sequence of *WsDWF5a* used for preparation of constructs for VIGS analysis is highlighted in yellow color.

Supplementary Table S1: Summary of 454 sequencing and assembly for leaf and root tissues of different chemotypes of *Withania somnifera*

Features	NMITLI-118		NMITLI-135		NMITLI-101[23]	
	Leaf	Root	Leaf	Root	Leaf	Root
HQ reads	675691	731352	708367	598182	834068	721755
Average HQ read length (bases)	311.23	282.28	292.75	287.97	311.63	282.28
Reads Assembled	589647	627707	619081	495622	640398	503970
Number of contigs	20621	22438	20135	18413	21445	20797
Average length of contigs (bases)	617.45	625.89	627.86	599.3	590.75	533.15
Range of contig length (bases)	100-10302	100-6249	100-5320	100-5191	100-5001	100-6724
Contigs above 200 bases	17819	19379	17342	15863	18515	17765
GC content	41.50%	41.20%	41.60%	41.33%	41.07%	40.48%
Percentage of large contigs	51.56%	52.30%	4.58%	4.83%	49.6 %	44.6 %
Average length of large contigs (bases)	917.96	925.64	889.01	922.05	885	821
Number of Singletons	63910	78645	66176	79737	68103	94017
Average length of singletons (bases)	208.29	280.08	265.27	264.28	280.64	259.61
Range of singleton length (bases)	50-602	50-712	50-954	50-588	50-602	50-612
Singletons above 200 bases	48988	59411	49663	58419	53115	69451
GC content	39.79%	39.10%	39.98%	40.36%	39.10%	38.19%

Supplementary Table S2: Features of combined assembly of transcriptomes of leaf and root tissues from different chemotypes of *W. somnifera*

**Features**	**Combined assembly**
HQ reads	573184 (193258119)
Reads Assembled	372999 (65.07%)
Number of contigs	43,287
Average length of contigs	617.05 bases
Range of contigs length	100-5278 bases
Contigs above 200 bases	36,372
GC content	40.38%
Percentage of large contigs	48.05%
Number Of Large Contigs	20,801
Average length of large contigs	973.17 bases
Number of Singletons	1,78,956
Average length of singletons	258.28 bases
Range of singleton lengths	50-1967 bases
Singletons above 200 bases	1,25,747
GC content	38.00%

Supplementary Table S3: Annotation summary of contigs and singletons from combined assembly against different databases

	**Total**	**TAIR10**	**NR**	**Tomato**	**Potato**	**CDD**
**Contigs**	43,287	23,116	24,897	27,010	25,872	20,104
**Singletons**	1,78,956	25,043	10,713	37,432	36,071	-
**Differentially expressed**	18,317	11,455	12,112	12,966	-	-

Supplementary Table S4: Numbers of unigenes encoding enzymes involved in triterpenoid backbone biosynthesis

Enzymes	Genes	Polypepetide (a.a.)	Alternatively spliced (intron length in bp)	Molecular Wt. (Kda)/pI
**Step1**				
**MVA pathway**				
Acetyl CoA acetyltransferase (AACT)	1	404	+ (79)	41.39/6.16
Hydroxymethyl glutaryl CoA synthase (HMGS)	2	462 462	-	51.16/6.09<br>51.04/6.20
3-hydroxy-3-methylglutaryl-coenzymeA reductase (HMGR)	5	575 600<br>599<br>602<br>575[12]	+ (881)	62.13/6.80<br>64.41/7.02<br>64.44/6.09<br>64.60/6.15<br>61.69/6.57
Mevalonate kinase (MK)	4	395<br>392<br>395<br>395	-	41.67/5.47<br>41.44/5.56<br>41.67/5.36<br>41.71/5.27
Phosphomevalonate kinase (PMK)	5	508<br>508<br>509<br>508<br>508	+ (1937)<br>+ (1254,1937)<br>+ (190)<br>+ (1937)<br>+ (1937)	55.03/5.44<br>55.07/5.52<br>55.52/5.82<br>55.05/5.52<br>55.14/5.95
Mevalonate diphosphosphate decarboxylase (MDD)	3	421<br>429<br>429	-	46.66/6.72<br>47.67/6.72<br>47.69/6.28
IPP diphosphate isomerase 1 (IPI)	1	290	+ (459, 215)	33.19/5.6
**MEP pathway**				
1-deoxy-D-xylulose-5-phosphate synthase (DXS)	2	717[14]<br>710	-<br>+ (56)	77.47/6.43<br>77.03/5.93
1-deoxy-D-xylulose-5-phosphate reductoisomerase (DXR)	1	475[14]	-	51.7/5.52
2-C-methyl-D-erythritol4-phosphate cytidylyl transferase (CDP-MES)	3	316<br>313<br>313	-<br>-<br>+ (235)	35.11/6.26<br>34.68/6.40<br>34.59/6.25

4-diphosphocytidyl-2-C-methyl-D-erythritol kinase (CDP-MEK)	1	409	-	45.23/6.61
2-C-methyl-D-erythritol 2,4-cyclodiphosphate synthase (MECPS)	Partial			
4-hydroxy-3-methylbut-2-enyldiphosphate synthase (HDS)	2	741	+ (85)	82.27/5.76
		741	+ (85)	82.26/5.80
4-hydroxy-3-methylbut-2-enyldiphosphate reductase (HDR)	3	460	+ (87)	51.75/5.67
		482	-	54.35/5.91
		461	-	51.83/5.70
**Step 2**				
Geranyl diphosphate Synthase (GPPS)	1	415	-	45.93/6.74
Farnesyl diphosphate Synthase (FPPS)	4	343	-	39.7/5.13
		342	+ (522, 424)	39.59/5.23
		342	-	39.54 /5.45
		343[13]	-	39.6/5.15
Squalene Synthase (SqS)	2	411	-	47.05/7.56
		411	-	47.06/7.92
Cycloartenol Synthase (CAS)	1	758	-	85.89/6.39
Cycloartenol C-24 methyltransferase (SMT1)	2	353	+ (117)	39.74/5.97
		346	+ (79, 140)	38.78/6.14
Sterol-4α-methyl oxidase 1 (SMO1)	2	300	-	34.96/7.69
		303	-	35.36/8.10
Cycloeucalenol cycloisomerase (CEC1)	Partial			
obtusifoliol 14-demethylase (CYP51G1)	2	487	-	55.33/8.67
		487	-	55.41/8.64
Δ14-sterol reductase (FK)	1	369	-	41.88/9.11
C-7,8 sterol isomerase (HYD1)	1	221	-	25.17/7.63
Sterol-4α-methyl oxidase 2 (SMO2)	1	269	-	31.24/7.74
C-5 sterol desaturase (STE1)	1	271	-	31.88/7.30
Sterol Δ7 reductase (DWF5)	2	434	+ (447)	49.75/9.02
		434	-	49.62/8.69

Supplementary Table S5: Total and differentially expressed unigenes annotated as members of gene families involved in secondary metabolite biosynthesis

Gene Families	Total unigenes	TAIR10	NR	Tomato	Differentially expressed	TAIR10	NR	Tomato
Cytochrome P450s	228	183	120	224	143	105	118	138
Glycosyltransferase	259	127	83	116	144	62	50	69
Methyltransferase	419	279	188	308	143	110	74	96
Transcription factors	1210	320	382	1023	624	491	561	624

Supplementary Table S6: Statistics of SSRs identified in contigs generated from assembly of leaf and root transcripts of three different chemotypes

	Leaf			Root		
	**NMITLI 118**	**NMITLI 135**	**NMITLI 101[a]**	**NMITLI 118**	**NMITLI 135**	**NMITLI 101[23]**
**Sequences examined**	20621	20135	21445	22438	18413	20797
**Number of identified SSRs**	514	470	729	636	414	703
**SSR containing sequences**	478	443	683	581	384	653
**Sequences with more than one SSR**	21	10	24	30	17	28
**SSRs present in compound formation**	47	25	86	64	36	95
**Di-nucleotide repeat**	166	142	175	202	124	177
**Tri-nucleotide repeat**	320	309	320	394	274	328
**Tetra-nucleotide repeat**	8	4	8	7	3	7
**Penta-nucleotide repeat**	0	0	3	8	0	7
**Hexa-nucleotide repeat**	15	15	11	19	12	15

Supplementary Table S7: Statistics of SSRs identified in singletons from leaf and root transcripts of three different chemotypes

	Leaf			Root		
	**NMITLI 118**	**NMITLI 135**	**NMITLI 101**	**NMITLI 118**	**NMITLI 135**	**NMITLI 101**
**Sequences examined**	63910	66176	68103	78645	79737	94017
**Number of identified SSRs**	1015	860	1824	1267	1049	2559
**SSR containing sequences**	939	806	1684	1193	985	2283
**Sequences with more than one SSR**	65	58	99	72	54	166
**SSRs present in compound formation**	129	107	226	140	107	461
**Di-nucleotide repeat**	473	396	541	597	492	647
**Tri-nucleotide repeat**	469	401	403	562	491	639
**Tetra-nucleotide repeat**	15	23	23	24	18	24
**Penta-nucleotide repeat**	11	8	14	10	8	15
**Hexa-nucleotide repeat**	20	14	26	31	26	20

Supplementary Table S8: List of different oligonucleotide primers for different genes used in this study

S. No.	Gene	Forward (5'-3')	Reverse (5'-3')
**For VIGS construct preparation**			
**1.**	**WsPDS**	TCTAGAGAGATTGTTATTGCTGGTGCAGG	CTCGAGAGGCACACCTTGCTTTCTCATCCA
**2.**	**WsDWF5a**	ATTGCCGAATTCCAGCAGCTACTCTCT	ACATAAGTGAATAAAGGAGCTCGTGCCAA
**For gene expression**			
**1.**	**WsPDS**	TGATGAGCTTTCAATGCAGTGC	CTTCCATCCGTATTCAGCTCAA
**2.**	**WsDWF5a**	ACGACGAACGGTGCAAGG	GCAGCAGCACTTATTAAAGAGCCA
**3.**	**WsTRV1**	TTACAGGTTATTTGGGCTAG	CCGGGTTCAATTCCTTATC
**4.**	**WsTRV2**	TTACTCAAGGAAGCACGATGAGC	GAACCGTAGTTTAATGTCTTCGGG
**5.**	**WsActin**	AGATATTCAGCCTCTTGTCTGTG	ATTGAGCCTCATCACCAACATA
**6.**	**WsHMGR**	CGCCGACCGACTCCTTGTCCTG	GAGAGGGAAGAGGTAGAGGAGGATC
**7.**	**WsMK**	GATCTAGAAACATGCGGATTCCAA	AGAATCAGGAATGCCGCTGTATA
**8.**	**WsFPPS**	TACTGATGATGCTAGTGAATGGGTCGAA	CACCAGCCGAGGGAAGATGTTTC
**9.**	**WsSQS**	CTCGGTCAAGGCAGTCCAATGTCTC	CACCATACACATCTGCCATAGTCCGA
**10.**	**WsSMO1**	GGTTATGCGTATGTTCATCCTCGTTG	TCCTCCACTATAAAGTACGTTCCCA
**11.**	**WsFK**	GAGGGAGAGAAGGGATGAAGCT	TTTACGGTATTCTGCCCAAACG
**12.**	**WsHYD1**	GTTCCCATATTCCTCTCACAGTCAG	GAACAACACAACCAGGACGGATGAG
**13.**	**WsDXS**	TGATGCAATTGAAAGTCCCATGAGCC	TTGAAGGTTTTTGGGCTTTTTTCATG
**14.**	**WsDXR**	GTCTATCAGAAGAAAGGAGCGGA	ATAGGCTTTGGACCATCCCAGC
**15.**	**WsCDPMEK**	TGGATAGGGAGGCTGGGCTTAC	TTGATGGCGACAGAGAGAACTT
**16.**	**WsCYP51G1**	ACCCTCCACTGATTATGCTTCTACGC	AAAATCTATCAGGGTCATAAGTATCTGGA
**17.**	**WsSTE1**	GTTCGTGGAAGAGACATCGTTTT	TAGACATTGCGCTTCAAGTGATAG
**18.**	**WsCAS**	CATCCTGGAAGAATGTGGTGCC	TCCTTTGCACACTCATTGCGAGC
**19.**	**TRVCP**	ATGGGAGATATGTACGATG	TAGGGATTAGGACGTATC
**20.**	**WsDWF5b**	GCATCAATGCTTACTCTCCTATCTTTCAC	ACCAGGCAATAGAAGTTGTAACGCT
